# Case Report: A Case of a Child With Facial Foreign Body Abscess and Facial Artery Pseudoaneurysm

**DOI:** 10.3389/fped.2022.886031

**Published:** 2022-04-28

**Authors:** Rong Kuang, Jing Zhou, Jiaqi Deng, Tian Xia, Mingxing Li

**Affiliations:** ^1^Department of Ultrasound, The Affiliated Hospital of Southwest Medical University, Luzhou, China; ^2^Department of Pathology, The Affiliated Hospital of Southwest Medical University, Luzhou, China

**Keywords:** traumatic, glass, foreign body, abscess, aneurysm, face

## Abstract

Facial artery pseudoaneurysms are rare and mostly a result of blunt injury. Since the facial arteries are well protected by facial soft tissue and the lumen of the facial artery is thin and small in diameter, a sharp injury usually leads to complete transection rather than partial laceration of the blood vessel. As a non-invasive method, ultrasound does not involve radiation and sedation. Diagnosis of facial artery pseudoaneurysms is most commonly made with ultrasound, and Doppler ultrasound is essential. On grayscale imaging, facial artery pseudoaneurysms often appearanced of a fluid collection, Color Doppler imaging often show a well-defined swirl pattern named “yin and yang sign,” the Spectral Doppler showed a diagnostic “to and fro” two-phase bidirectional arterial blood flow spectrum. It’s particularly for the examination of facial artery pseudoaneurysms in children. Here, we report a case of facial foreign body abscess and facial artery pseudoaneurysm in a 19-month-old child 1 week after a sharpness injury that was diagnosed by ultrasound.

## Case Report

**FIGURE 1:**
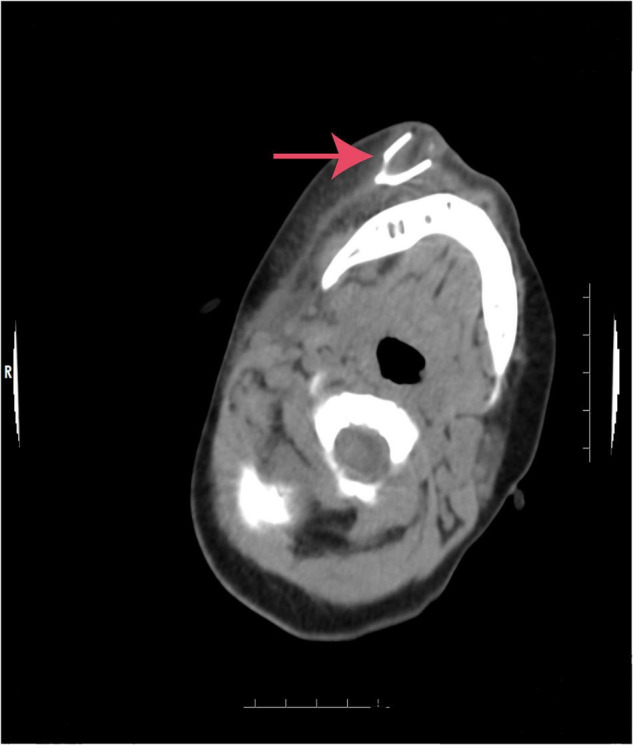
Computerized tomography (CT) scan of the head and face. A short strips of “U” shaped high density structure (red arrow) due to foreign body in the right cheek.

**FIGURE 2:**
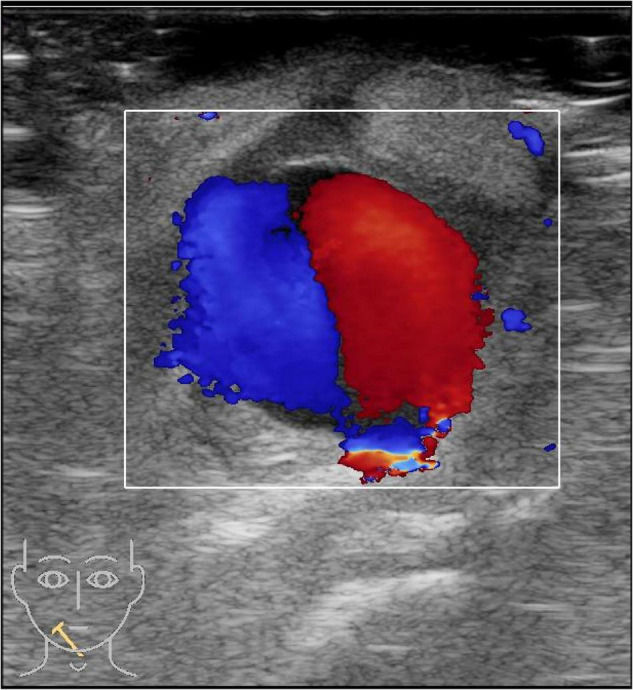
A color Doppler ultrasound of the right cheek. The red and blue blood flow signals are shown as “yin and yang sign”.

**FIGURE 3:**
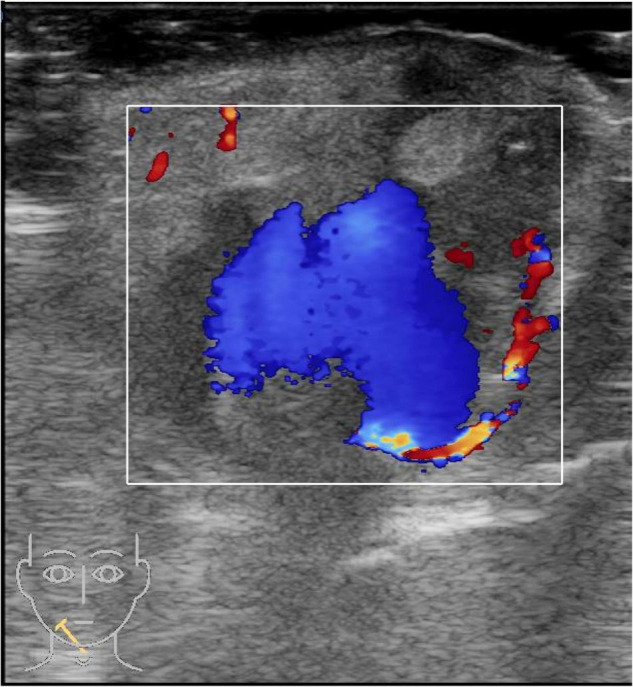
A color Doppler ultrasound of the right cheek. The pseudoaneurysm is connected to a branch of the facial artery.

**FIGURE 4:**
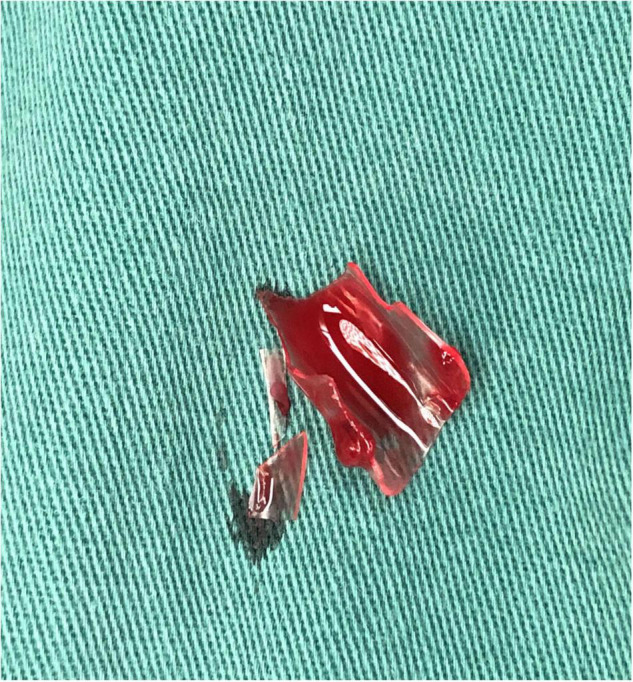
Surgical specimen. A ring-shaped glass bottle with a length of about 1.2 cm and a diameter of 0.5 cm and three pieces of rice-sized glass slag were removed during the operation.

**FIGURE 5:**
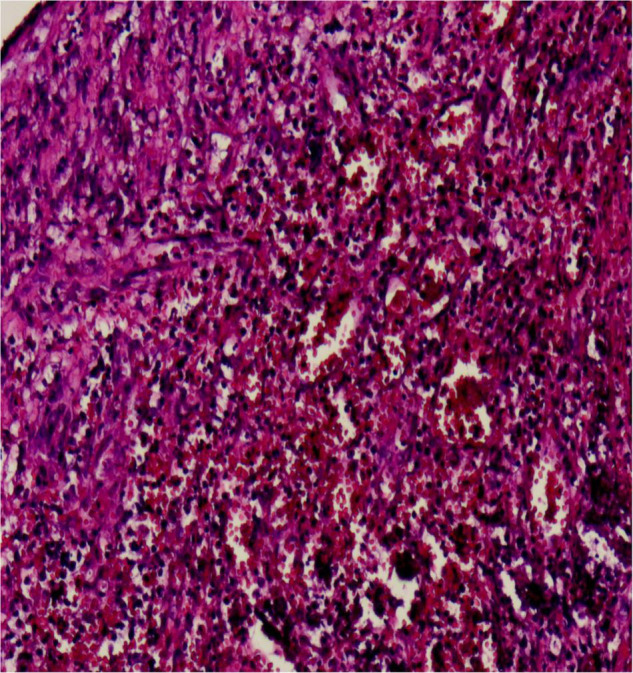
Microscopic image of the lesion (hematoxylin and eosin staining, magnification ×200). Showing the tumor wall was composed of mixed thrombus with organization.

A 19-month-old girl who suffered from pain and bleeding on the right cheek due to a puncture from a glass bottle was presented to the local hospital. She was treated with local anesthesia and debridement and suturing. One week later, the suture was removed. One week after the suture removal, the child had redness and swelling on the right cheek wound which was aggravated for 3 days. Upon physical examination, the right cheek was red and swollen, the skin temperature was high, and a black necrotic area with a diameter of about 0.5 cm was seen in the center. The surface was palpable and throbbing. When squeezed, the wound was painful and oozed pus. In addition, systolic murmurs could be heard on auscultation. A computerized tomography (CT) scan of the head and face (plain scan) showed a short strips of “U” shaped high density structure (red arrow) due to foreign body in the right cheek space with a diameter of about 1.8 cm (Figure 1). The formation of a foreign body in the right cheek space with local hematoma was considered. Upon ultrasound examination, an irregular dark area was seen on the right cheek, a “C” type strong echo was seen in the dark area, and color Doppler flow imaging (CDFI) showed no blood flow signal in the dark area. Additionally, a “C” type strong echo was seen in the dark area immediately adjacent to the irregular dark area, and a round cystic mass with a diameter of about 1.7 × 1.7 cm was seen on the deep surface with clear boundaries and obvious pulsation. CDFI showed the red and blue blood flow signals are shown as “yin and yang sign” (Figure 2), which was continuous with a branch of the facial artery (Figure 3). PW showed the “bi-phase bidirectional” arterial blood flow spectrum at the breach. Based on these findings, the patient was diagnosed with a foreign body on the right cheek with surrounding abscess and facial artery pseudoaneurysm. Surgery was performed immediately. A ring-shaped glass bottle with a length of about 1.2 cm and a diameter of 0.5 cm and three pieces of rice-sized glass slag were removed during the operation (Figure 4). A pseudoaneurysm with a diameter of about 1.8 cm was seen on the deep side next to the glass foreign body, and the tumor was ligated and excised. Pathological analysis revealed that the tumor wall was composed of mixed thrombus with organization (Figure 5). The patient was followed up after the operation, and the wound healed well.

## Discussion

About 85% of craniofacial pseudoaneurysms involve the superficial temporal artery because the artery passes through the frontal bone between the temporal and frontal muscles and lacks muscle tissue protection. Other arteries that can be involved include the maxillary artery and the facial artery. Maxillary artery pseudoaneurysm is usually caused by iatrogenic injury, and facial artery pseudoaneurysm is often secondary to facial blunt injury (1). Facial arteries are protected by facial soft tissues. They have a thin lumen and small diameter (about 1.3–2.2 mm). Sharpness damage usually leads to a complete transection; thus, part of the vessel wall rupture is rare (2). Previously reported injury factors include sudden trauma, gunshot wounds, glass, intraoral stents, infections, etc. (3–5). In this case, the pseudoaneurysm was close to the foreign body, the abscess surrounded the upper part of the pseudoaneurysm, and the pulsatile mass on the face appeared after suture removal; so risk factors included iatrogenic injury, glass foreign body injury, and infection, which have not been reported previously. The diagnosis mainly included clinical manifestations such as pulsating masses and systolic murmurs and imaging examinations. Magnetic Resonance Angiography (MRA) and Computed Tomography Angiography (CTA) can comprehensively display the shape and branch of the facial artery while excluding more extensive lesions (6), but this latter requires radiation and needs to be braked during the inspection, so it has relatively poor practicality in children. Ultrasonography is the best modality to assess these pseudoaneurysms (7), as a non-invasive examination method, requires no radiation and the patient does not need to remain stationary; thus, has diagnostic value for pseudoaneurysms, particularly for the detection of facial artery pseudoaneurysms in children (8, 9). In our case, the grayscale imaging showed an anechoic zone with weak and echogenic light spots rotating and fluttering, thrombus attachment on the wall, and pulsatile tubular anechoic on the periphery connected to the dark zone. Color Doppler is useful for the diagnosis, to evaluate the neck of pseudoaneurysm and to trace the feeding artery (10). The color Doppler showed a systolic breach with bright and colorful; the blood flow in the tumor body was red and blue depicting the “yin and yang sign” due to turbulent internal flow (11). Chen et al. suggested that the “yin and yang sign” is diagnostic of a pseudoaneurysm (12). Lastly, the spectral Doppler showed a diagnostic “to and fro” two-phase bidirectional arterial blood flow spectrum. This occurs due to blood flowing into the tumor body during systole and out of the tumor body during diastole. Ultrasound provides excellent detection and characterization of soft tissue foreign bodies. According to the foreign body biological characteristics of soft tissues, they are hyperechoic to varying degrees on ultrasound and similar in shape to the foreign body morphology. High frequency linear array transducers are the most useful in detection of radiopaque and non-radiopaque Soft tissue foreign bodies (13). The utility of ultrasound can also be applied intraoperatively, allowing the surgeon to locate the foreign body in real-time. In the case of a false facial artery aneurysm, facial artery ligation is the preferred treatment method as it has the advantage of a simple operation and low complication rates (3, 4).

## Conclusion

We suggest that it is necessary to examine and follow up the wound site after an acute facial injury. Ultrasound should be used as a non-invasive, safe, and effective method for the diagnosis of facial artery pseudoaneurysm.

## Ethics Statement

Ethics review and approval/written informed consent was not required as per local legislation and institutional requirements.

## Author Contributions

RK wrote all drafts. JZ discussed the meaning of the draft. JD collected all the references. TX carried out the pathology and collected the clinical data. ML offered conception and finalized the draft. All authors read and approved the final manuscript.

## Conflict of Interest

The authors declare that the research was conducted in the absence of any commercial or financial relationships that could be construed as a potential conflict of interest.

## Publisher’s Note

All claims expressed in this article are solely those of the authors and do not necessarily represent those of their affiliated organizations, or those of the publisher, the editors and the reviewers. Any product that may be evaluated in this article, or claim that may be made by its manufacturer, is not guaranteed or endorsed by the publisher.
